# Navigating Glioblastoma Diagnosis and Care: Transformative Pathway of Artificial Intelligence in Integrative Oncology

**DOI:** 10.7759/cureus.44214

**Published:** 2023-08-27

**Authors:** Muhammad Ibrahim, Quratulain Muhammad, Aroosa Zamarud, Hadia Eiman, Faizan Fazal

**Affiliations:** 1 Department of Medicine, Rawalpindi Medical University, Rawalpindi, PAK; 2 Department of Neurosurgery, Stanford Health Care, Palo Alto, USA

**Keywords:** grade iv glioma, ai in robotics and healthcare, radiomics, artificial intelligence, glioblastoma multiforme

## Abstract

Glioblastoma multiforme (GBM), an aggressive brain tumor with high recurrence rates and limited survival, presents a pressing need for accurate and timely diagnosis. The interpretation of MRI can be complex and subjective. Artificial Intelligence (AI) has emerged as a promising solution, leveraging its potential to revolutionize diagnostic imaging. Radiomics treats images as numerical data and extracts intricate features from images, including subtle patterns that elude human observation. By integrating radiomics with genetics through radiogenomics, AI aids in tumor classification, identifying specific mutations and genetic traits. Furthermore, AI's impact extends to treatment planning. GBM's heterogeneity and infiltrative growth complicate delineation for treatment purposes. AI-driven segmentation techniques provide accurate 2D and 3D delineations, optimizing surgical and radiotherapeutic planning. Predictive features like angiogenesis and tumor volumes enable AI models to anticipate postop complications and survival rates. It can also aid in distinguishing posttreatment radiation effects from tumor recurrence. Despite these merits, concerns linger. The quality of medical data, transparency of AI techniques, and ethical considerations require thorough addressing. Collaborative efforts between neurosurgeons, data scientists, ethicists, and regulatory bodies are imperative for AI's ethical development and implementation. Transparent communication and patient consent are vital, fostering trust and understanding in AI-augmented medical care. In conclusion, AI holds immense promise in diagnosing and managing aggressive brain tumors like GBM. Its ability to analyze complex radiological data, integrate genetics, and aid in treatment planning underscores its potential to transform patient care. However, carefully considering ethical, technical, and regulatory aspects is crucial for realizing AI's full potential in oncology.

## Editorial

Glioblastoma multiforme (GBM), the most common primary adult CNS malignancy, is classified as a grade IV glioma by the World Health Organization (WHO). With an incidence of 3.2 per 100,000 population, it is the most aggressive brain tumor with a guarded prognosis owing to its invasive nature and a high recurrence rate of about 90% [1]. All these grim features reduce the median survival to almost a year and the five-year survival rate significantly to about 5%. These considerations call for an early and accurate diagnosis, an essential part of managing all GBM cases. The gold standard imaging technique for GBM is magnetic resonance imaging (MRI). It is a non-invasive tool that offers good diagnostic efficacy; however, its interpretation can be complex, time-consuming, and subjective. In some instances, it may remain inconclusive. Since malignancies are defined based on imaging combined with histopathology and genomics, there is a need to accurately interpret and interlink these diagnostic modalities for a reliable diagnosis.

Artificial Intelligence (AI) is a burning topic of debate nowadays, envisioned to revolutionize healthcare and diagnostic imaging by its ever-expanding potential. AI is a vast term that incorporates natural language processing, machine learning, and deep learning to formulate an integrated and fully automated system, which has the potential to act as an ancillary in the effective management of patients in the healthcare setting [1,2]. There is growing interest in the application of AI in oncology and neuroimaging, which can overpower traditional diagnostic algorithms. Machine learning algorithms are able to identify and interlink numerous imaging features that are subtle for the human eye to distinguish, consequently increasing the accuracy of the grading prediction. Within this resides the ultimate goal of maximizing the potential of AI to help clinicians in the diagnosis and minimize the stress of invasive procedures. Herein, we discuss the potential of AI tools in diagnosing aggressive brain tumors with a particular focus on GBM, emphasizing the role of AI in neuroradiology.

The efficacy of AI-powered tools has been demonstrated in detecting glioblastomas and differentiating them from small brain metastasis and primary CNS lymphomas, which appear quite similar on MRI but have very different treatment modalities [2]. These tools use radiomics, which treats radiological images as numerical data and extracts quantitative information, such as size, shape, position, texture, and intensity, from images in addition to complex patterns, such as peri-tumoral characteristics, which are not usually picked by the human eye. The radiomic approach also complements histological assessment. AI tools have been used to grade gliomas such as low-grade gliomas vs. high-grade gliomas. Radiogenomics is a branch of radiomics that combines images (the phenotype) with genetics (the genotype) in classifying tumors. In recent years, several studies have been done to explore the potential of machine learning tools in classifying tumors into isocitrate dehydrogenase (IDH) wild type vs. IDH mutated type gliomas, O(6)-methylguanine-DNA-methyltransferase (MGMT) promoter mutated gliomas. Similarly, AI tools have been shown to detect 1p19q codeletion, which is present in oligodendroglioma and EGFR mutations, characteristic of GBM [2,3]. In addition to accurately diagnosing GBM, AI tools also play an essential role in determining the treatment approach. GBM has diffuse infiltrative growth and heterogeneity, making delineation and treatment planning difficult. Deep learning helps delineate regions of interest in 2D and 3D, i.e., segmentation that is crucial for treatment planning. Segmentation aims to maintain an onco-functional balance; too aggressive resection can lead to reduced quality of life and being too cautious can lead to an increased risk of recurrence after surgery or radiotherapy. At present, convolution neural networks (CNNs), which is a subset of deep learning tools. are the most reliable for segmentation with a reported accuracy of 90% [2].

AI-based tools can use predictive features, such as angiogenesis, tumor volumes, peritumoral infiltration, distance to the ventricles, tumor hypoxia, and cell density, to predict postop complications and survival. However, not much work has been done in this regard. AI models have shown reliable accuracy in differentiating post-treatment radiation effects (PTRE), such as pseudo-progression or radiation necrosis from true progression, which has always been a diagnostic dilemma for radiologists. The accurate distinction of recurrence from pseudo-progression helps in optimizing chemotherapy protocols in post-surgical GBM patients [3]. Most described algorithms had accuracy levels above 80-90% [4]. These features have the additional advantage of significantly lowering the workload of healthcare staff. By using AI in areas of diagnosis, healthcare manpower could be utilized in other important aspects of patient care. This has the potential to solve the problem of healthcare staff shortage that is being faced worldwide. And indeed, all of these will ensure better patient care and improved patient satisfaction levels.

Certain features of AI have concerned clinicians. One significant obstacle to the efficient growth of AI applications in oncology is data quality. AI models require large amounts of high-quality and labeled data, whereas medical data are often fragmented and unlabeled. This leads to problems such as bias, improper curation, and low reliability [3,5]. Bias might be introduced when the AI models are trained on data that are not representative of the target patient population due to differences in genetics, healthcare systems, and socioeconomic conditions. For example, if a skin cancer detection model is primarily trained on images of lighter skin tones, it may develop an algorithm that will perform poorly on darker skin tones, leading to misdiagnosis. Second, the lack of transparency regarding AI techniques is a significant concern. Any medical care system needs to be understandable and explicable in order to win the trust of physicians, administrators, and patients. It should ideally be able to fully explain to all parties concerned the reasoning behind a decision. Third, patients and doctors naturally view the incorporation of AI with skepticism due to the traditional diagnosis and treatment models' strong hold on the public. Consent, data anonymization, and de-identification are other privacy and data protection issues that garner ethical concerns. In addition to technology, ethical, intellectual, moral, and economic concerns must be addressed for intelligent oncology to grow smoothly [5]. It is imperative to note that AI tools should always act as auxiliary aids to a physician. At the current level of development, they cannot be used as autonomous decision-making tools.

The only way forward is interdisciplinary collaboration. Neurosurgeons, data scientists, AI experts, ethicists, and regulatory bodies must collaborate to form robust guidelines and standards for the ethical deployment of AI tools in patient care. The development of explainable AI models is necessary to overcome the lack of transparency in decision-making algorithms. Taking informed consent from patients is vital, as they deserve to understand how AI influences their medical care and outcomes. More research needs to be done to expand the horizons of AI, advancing its capabilities and limiting its restrictions, especially in the context of GBM diagnosis and management. Lastly, proper tuning and rigorous real-life testing of AI tools are necessary before introducing them to ensure diagnostic accuracy.
